# Herbal formula xuling-jiangu improves bone metabolic balance in rats with ovariectomy-induced osteoporosis via the gut-bone axis

**DOI:** 10.3389/fphar.2024.1505231

**Published:** 2024-11-13

**Authors:** Juan Chen, Szetuen Ng, Pengchao Xu, Sainan Chen, Shengqiang Li, Xuan Chen, Lihua Xie, Jirong Ge

**Affiliations:** ^1^ Fujian Key Laboratory of Integrated Traditional Chinese and Western Medicine for the Prevention and Treatment of Osteoporosis, Fujian Academy of Chinese Medical Sciences, Fuzhou, China; ^2^ Department of Orthopedics and Traumatology, Fujian University of Traditional Chinese Medicine, Fuzhou, China

**Keywords:** XuLing JianGu recipe, osteoporosis, metagenomic, metabolomic, gut-bone axis

## Abstract

**Introduction:**

The XuLing JianGu recipe (XLJGR) is an empirical traditional Chinese medicine formula used for the treatment of osteoporosis. This study aims to explore the effects of XLJGR on the intestinal microbiota composition and endogenous metabolites in ovariectomized (OVX) rats.

**Methods:**

An OVX rat model was established to evaluate the intervention effects of XLJGR. The measured indicators included bone density, serum bone metabolism markers, and an analysis of the types and abundances of intestinal microbiota, along with changes in endogenous metabolites. Additionally, MC3T3-E1 cells were used to validate the differential metabolites.

**Results:**

XLJGR significantly reduced the abundance of Bacteroides, Butyricicoccus, and other bacterial strains in the gut. KEGG metabolic pathway enrichment analysis showed that XLJGR intervention led to notable changes in pathways such as peptidoglycan biosynthesis, carbapenem biosynthesis, and vancomycin resistance. Moreover, XLJGR significantly upregulated key intestinal microbiota metabolites, including gabapentin(GAB), camphoric acid(CAA), and nonanedioic acid(AZA), thereby promoting the proliferation and osteogenic differentiation of MC3T3-E1 cells.

**Discussion:**

This study highlights the potential biomedical applications of XLJGR in promoting bone health by positively affecting intestinal microbiota and metabolic characteristics. These findings suggest that XLJGR may serve as a viable alternative in the treatment of osteoporosis, warranting further exploration of its therapeutic mechanisms and clinical applications.

## 1 Introduction

Osteoporosis (OP) is a systemic metabolic bone disease that is characterized by decreased bone mass, destruction of bone tissue microstructure, reduced bone strength, and increased risk of fractures o (Huidrom et al., 2021). With the aging global population, the incidence of age-related diseases is gradually rising. OP has become a significant health issue for middle-aged and elderly individuals, particularly in China (Wang et al., 2021). Therefore, the effective prevention and treatment of OP are of utmost importance for the health and wellbeing of the elderly. Current chemical treatments for OP often come with adverse reactions, and the high cost of medications may limit their accessibility and affordability (Rossini et al., 2016). Traditional Chinese Medicine (TCM) may serve as a valuable complement to existing interventions, especially among high-risk women, where its usage remains relatively low (Lee et al., 2022; Wang et al., 2017). However, the complex components and unclear mechanisms of action in TCM may pose certain limitations.

Many studies have shown that intestinal microflora plays a key role in various metabolic diseases, and other literature has reported its association with the development of OP (Iatcu et al., 2021; Chu et al., 2021). Intestinal microbiota can regulate bone mineral density (BMD) by affecting the immune system intestinal barrier function, calcium absorption, estrogen levels and so on. The species and abundance of intestinal microflora serve as the material basis for their influence on the balance of bone metabolism through multiple pathways, including bone immunity (Seely et al., 2021; Weersma et al., 2020). In 2000, Arron proposed the concept of “bone immunology” (Arron and Choi, 2000). With the deepening of research, the intricate reciprocal regulatory mechanism between the immune system and bone has become increasingly recognized. The immune cells and their secreted cytokines have important effects on bone remodeling. Furthermore, gut microbiota enhance the absorption of minerals such as calcium and release substances that facilitate mineral uptake. Their metabolic products, including short-chain fatty acids, promote the proliferation of osteoblasts while inhibiting the activity of osteoclasts, and they also influence the production of cytokines related to bone metabolism (Seely et al., 2021; Lu et al., 2021; Sergio et al., 2022).

XuLing JianGu recipe (XLJGR) is an empirical traditional Chinese medicine formula developed by our research team for the treatment of osteoporosis, which has been granted a Chinese national patent. In our previous work, we found that XLJGR could improve BMD and biomechanics in model rats, affect the metabolic balance of calcium and phosphorus, and modulate the function of the OPG/RANK/RANKL signaling pathway (Ge et al., 2016). In this study, the effects of XLJGR on intestinal flora and endogenous metabolite changes were determined through the integrated metagenomic and metabolomic analysis, so as to further elucidate the gut microbiota-bone mechanism of XLJGR in treating osteoporosis. These findings suggest that XLJGR has significant advantages in biomedical applications and may serve as a strong alternative to existing treatment methods, providing new perspectives and options for the treatment of OP.

## 2 Methods

### 2.1 Experimental animals and osteoporosis model

Thirty 3-month-old SPF level Sprague-Dawley (SD) female rats were supplied by Shanghai Slake Laboratory Animal Co., Ltd. (Shanghai, China; Certificate No. SCXK 2007-0005). The rats were housed in the Experimental Center of Comparative Medicine of Fujian Academy of Chinese Medical Sciences (Fujian, China; Certificate No. SYXK 2016–0005). All animals were maintained under the same conditions in an environment where the room temperature, relative humidity and light cycle were 19°C ± 2°C, 58% ± 12% and 12-h/12-h, respectively. Common feed, free water and activities were provided. This study was approved by the Animal Ethics Committee of Fujian Academy of Chinese Medical Sciences (approval number: FJATCM-IAEC2018034).

The rats were randomly divided into three groups: sham operation (Sham) group, osteoporosis model (OP) group and Xu-Ling-Jian-Gu recipe (Xu) group, with 10 rats in each group. The animal model was constructed by classical ovariectomized (OVX) model, except the Sham group. All rats were anesthetized with 10% chloral hydrate via intraperitoneal injection and fixed on their backs. Under sterile conditions, the abdomen was cut in the middle, the ovaries of both sides were removed in both OP and Xu groups, while the adipose tissues of similar size near the ovaries were removed in Sham group. To prevent infection, each rat was given an intraperitoneal injection of 20,000 U/100 g penicillin daily for 3 days after surgery.

### 2.2 Preparation and identification of experimental herbs

XLJGR consists of 12 traditional Chinese medicines, including XuDuan, FuLing, BaiZhu, HongHua, ChiShao, GanCao, ChenPi and so on (see Supplementary Material, Supplementary Table S1 for detailed composition). The total weight of each dose of dried medicinal herbs is 122 g. Five doses of XLJGR were chosen, soaked in 10 times the amount of water (g/v) for 30 min.Three batches of XLJGR were prepared in parallel using the above-mentioned herbal preparation method to identify the active ingredients. Each batch underwent two tests. The detection instrument was Rigol L3000 high performance liquid chromatography equipped with Rigol C18 reverse phase column (250 mm × 4.6 mm, 5 µm).

### 2.3 Drug intervention and collecting sample

Drug intervention was started 30 days after modeling. The rats in Xu group were administered an intragastric dose of 10 mL/kg, while those in OP and Sham groups were given the same amount of 0.9% normal saline by gavage. The drug was administered once a day for a duration of 12 weeks.

All rats were anesthetized 12 h after the last gavage, and the abdominal area of each rat was sterilized with 75% ethanol. The whole blood remained static for 2 h, and then centrifuged at 4,000 r/min for 15 min. The left tibia of all animals was collected, and the soft tissues surrounding it were cleared. Fresh stool samples were collected from rats in each group using the abdominal compression method, and then stored at −80°C for metagenomic sequencing.

### 2.4 Bone mineral density (BMD) tests

The BMD of proximal tibia in small animal model was measured by dual-energy X-ray absorptiometry (HOLOGIC, Discovery WS/N89006).

### 2.5 Enzyme-linked immunosorbent assay (ELISA)

CTX-1 and P1NP are selected as bone metabolism biomarkers because CTX-1 reflects osteoclast activity and P1NP indicates osteoblast function. Their combined use offers a clearer balance of bone turnover, making them reliable indicators for assessing osteoporosis compared to other biomarkers (Schini et al., 2023).The serum was thawed at 4°C, diluted, and then analyzed using the Rat CTX-1 (CUSABIO, CSB-E12776r) and P1NP (CUSABIO, CSB-E12774r) ELISA Kits according to the manufacturer’s instructions. The OD value was detected at 450 nm using a microplate reader, and a standard curve was drawn to calculate the sample concentration.

### 2.6 Metagenomic sequencing

Total DNA of the microorganisms was extracted using the CTAB method. The DNA sequence was randomly fragmented into short fragments ranging from 200 to 500 bp. After passing the quality inspection, high-throughput sequencing was performed using the NovaSeq 6,000 system in PE150 mode. After obtaining effective data, CDS was predicted using the Meta Gene Mark (v3.26). The data were then clustered and redundancy was removed by CD-HIT (v4.6.1). GO and KEGG databases were used for annotation and enrichment analysis of the gene sets. CapitalBio Technology was entrusted to complete the metagenomics sequencing for this study.

### 2.7 Metabolomics analysis of rat plasma

The plasma was thawed at 4°C, and 100 µL of each plasma sample was added to 300 µL acetonitrile to precipitate proteins. After vortexing for 5 min, the supernatant was centrifuged at 12,000 rpm for 5 min. After being separated with high performance liquid chromatography (HPLC), the samples were analyzed by mass spectrometry under positive and negative electrospray ionization modes. Compound Discover V3.1 software was used for data extraction and processing.

### 2.8 Intervention with differential metabolites in MC3T3-E1 cells

According to the metabolomics results, different concentrations of gabapentin, camphoric acid and azelaic acid were used to intervene in MC3T3-E1 cells. MC3T3-E1 cells were seeded in 96-well plates at a density of 5,000 cells per well. After 24, 48 and 72 h of intervention, cell count kit-8 (HY-K0301-500T) was performed, and the OD values were determined at 405 nm. Alkaline phosphatase (ALP) activity was detected using an ALP calcium.

### 2.9 Statistical analysis

SPSS 25.0 software was used for data analysis, and the measurement data were expressed as mean ± standard deviation. *t*-test analysis was conducted to determine the significance of inter-group differences in metabolites. For multi-group comparison, the data with uniform variance of normal distribution were analyzed by one-way analysis of variance (ANOVA).

## 3 Results

### 3.1 Identification of effective components in XLJGR

**FIGURE 1:**
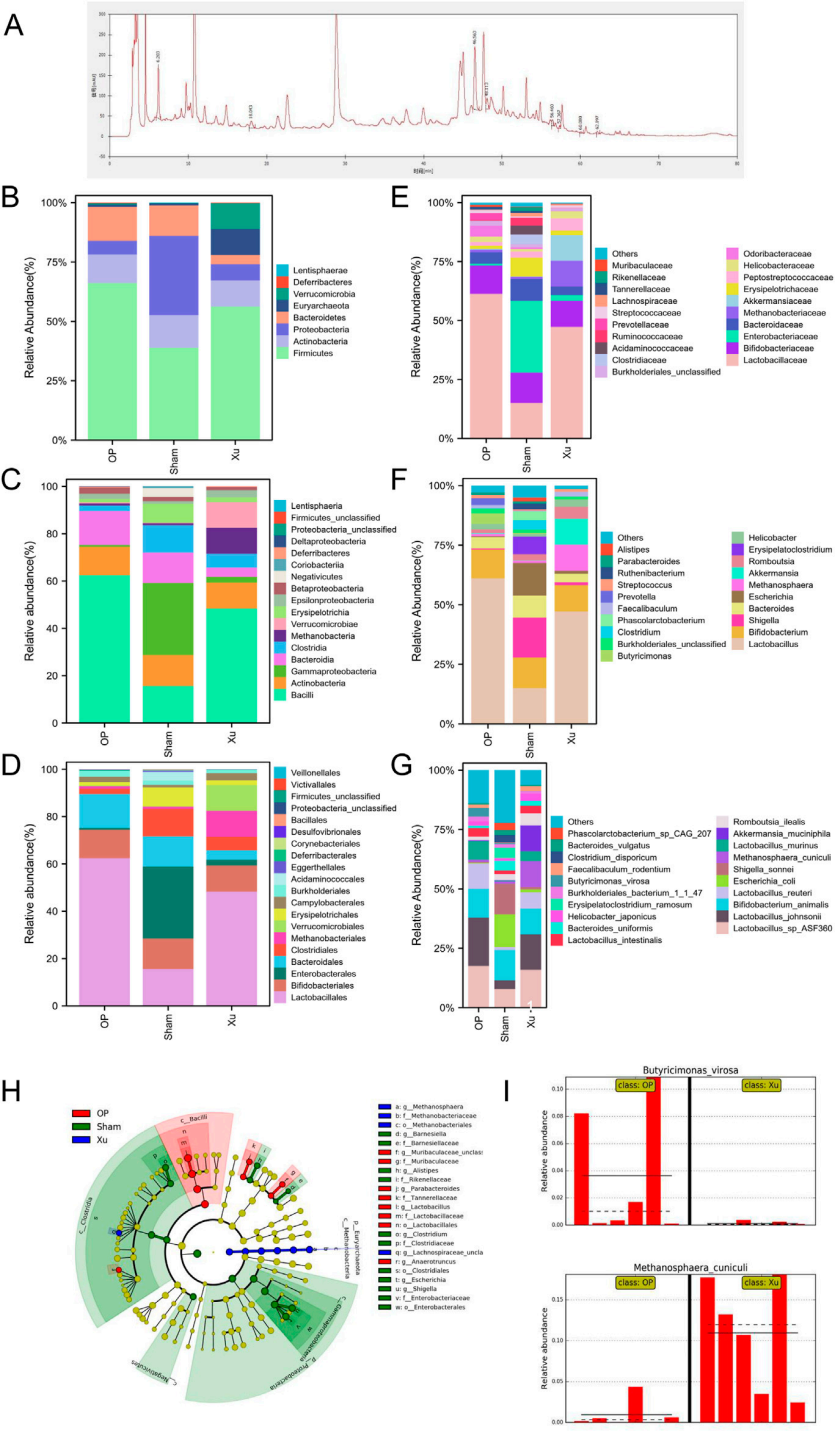
Differences in relative abundance of intestinal microbiota among groups. **(A)** HPLC chromatogram of XLJGR. **(B)** Intestinal microbiota at the taxonomic levels of phylum. **(C)** Intestinal microbiota at the taxonomic levels of class. **(D)** Abundance of intestinal microbiota at the taxonomic levels of order. **(E)** Intestinal microbiota at the taxonomic levels of family. **(F)** Intestinal microbiota at the taxonomic levels of genu. **(G)** Abundance of intestinal microbiota at the taxonomic levels of species. **(H)** The cladogram of intestinal microbiota abundance in groups. **(I)** Differences at the phylum level analyzed by Galaxy.

Ten effective components were identified from XLJGR via HPLC analysis, including gallic acid, hydroxy safflower yellow pigment A, paeoniflorin, ferulic acid, naringin, tetrahydropalmatine, dehydrogenated pachymic acid, atractylone, dipsacoside VI and paeonol. The spectra of both standard and samples are displayed in Figure 1A, and the detailed information of effective components is shown in Supplementary Table S2.

### 3.2 Effect of XLJGR on BMD and serum markers of bone metabolism in OVX rats

The BMD of the left proximal tibia is shown in Supplementary Table S3. The results demonstrated that BMD was decreased in OP group compared with Sham group (P < 0.05). Moreover, BMD was higher in Xu group than in OP group (P < 0.05).The results of rat serum CTX-1 and P1NP are shown in Supplementary Table S4. CTX-1 and P1NP were decreased in OP group compared with Sham group (P < 0.05). However, there was no difference in CTX-1 between the Xu and OP groups. P1NP showed an increasing trend in XU group when compared to OP group (P > 0.05).

### 3.3 Effect of XJLGR on intestinal microbiota in OVX rats

The MetaPhlAn2 module of Galaxy software analyzed differences in classified strains and the impact of XLJGR on their abundance. Figure 1 illustrates the relative abundance of three bacterial groups across six taxonomic levels: phylum, class, order, family, genus, and species. At the phylum level, the OP group showed increased Firmicutes and Bacteroidetes compared to the Sham group, with a Firmicutes/Bacteroidetes (F/B) ratio of 4.61. XLJGR reduced these phyla’s abundance and raised the F/B ratio to 14.40, while increasing Verrucomicrobia and Euryarchaeota. At the class level, Bacteroidia and Bacilli were higher in the OP group, but XLJGR decreased their abundance. XLJGR also boosted Verrucomicrobiae and Methanobacteria levels. At the order level, Lactobacillales and Bacteroidales increased in the OP group, but XLJGR reduced them and increased Verrucomicrobiales and Methanobacteriales. Similar trends were observed at the family and genus levels, with XLJGR decreasing Lactobacillaceae and Butyricimonas while increasing Akkermansia and Methanosphaera. Species-level differences were marked by higher Akkermansia_muciniphila and Methanosphaera_cuniculi in the Xu group, and *Shigella*_sonnei and *Escherichia*_coli in the Sham group. The LEfSe module further analyzed differential flora, revealing significant differences in Euryarchaeota and Proteobacteria, with Butyricimonas_virosa and Methanosphaera_cuniculi showing the most pronounced distinctions.

### 3.4 Effect of XLJGR on intestinal microbiota function in OVX rats

**FIGURE 2:**
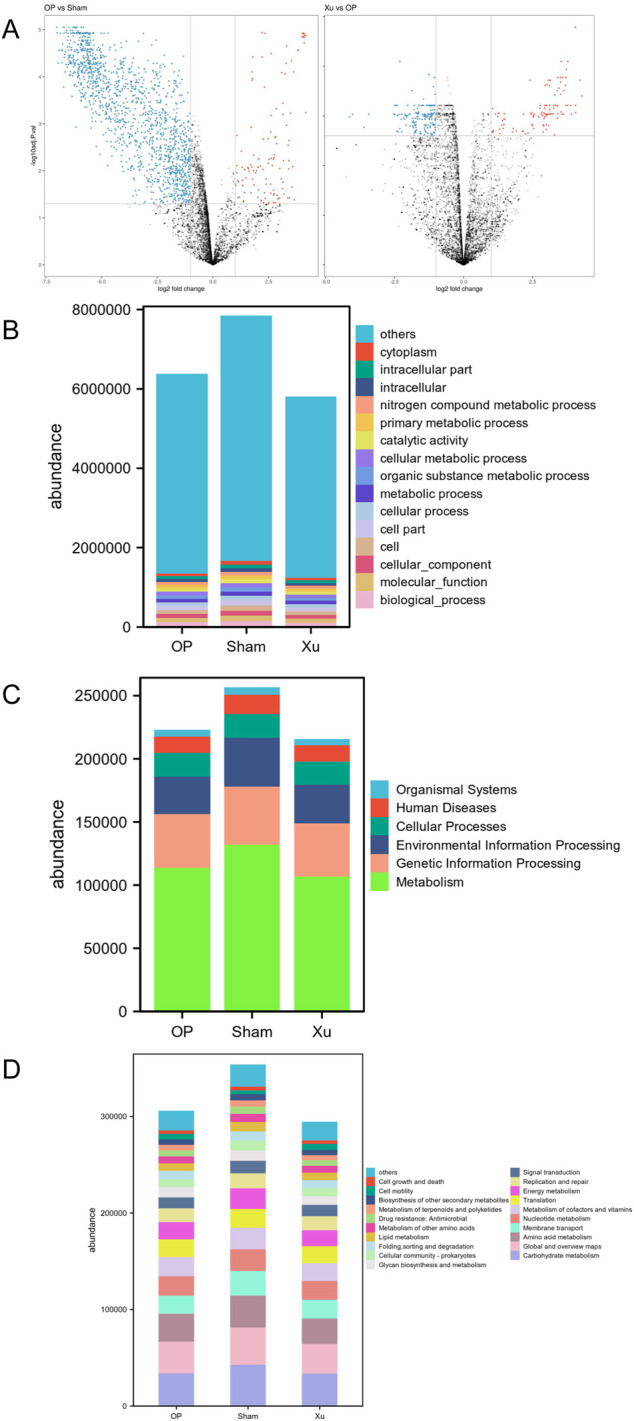
GO and KEGG functional annotation and enrichment analysis of differential genes in the intestinal microflora. **(A)** Volcano map between groups in GO analysis. **(B)** GO functional annotation and enrichment analysis of differential genes in the intestinal microflora among groups. **(C)** KEGG Level 1 functional annotation and enrichment analysis of differential genes in the intestinal microflora among groups. **(D)** KEGG Level 2 functional annotation and enrichment analysis of differential genes in the intestinal microflora among groups.

Principal coordinates analysis (PCoA) was performed on the functional genes obtained from GO annotation and the metabolic pathways derived from KEGG enrichment in gut microbiome samples. Differences between the two groups were analyzed using the Bray-Curtis dissimilarity matrix and multivariate analysis of variance (Adonis). The results indicated that the functional gene differences between the Sham and OP groups were statistically significant (R^2^ = 0.5887, *p* = 0.0021) (Figure 2A). A total of 2,377 genes were identified, with 2,109 genes upregulated in the Sham group and 268 genes upregulated in the OP group. Furthermore, significant differences in functional genes were observed between the OP and Xu groups (R^2^ = 0.2514, P = 0.0016), with 450 genes showing statistical significance. GO functional annotation and enrichment analysis revealed the highest abundance of biological processes (BP) among the three groups (Figure 2B). At the first level of KEGG, the three groups were enriched in six major biological metabolic pathways, with metabolic pathways accounting for over 50% (Figure 2C). At the second level, carbohydrate metabolism had the highest proportion among the subfunctions, and the top 20 pathway subfunctions included various metabolism-related processes such as amino acid and nucleotide metabolism, cofactor and vitamin metabolism, and energy metabolism (Figure 2D). Differentially expressed genes and pathways are detailed in Supplementary Tables S5, S6.

### 3.5 Effect of XJLGR on endogenous metabolites in plasma of OVX rats

**FIGURE 3:**
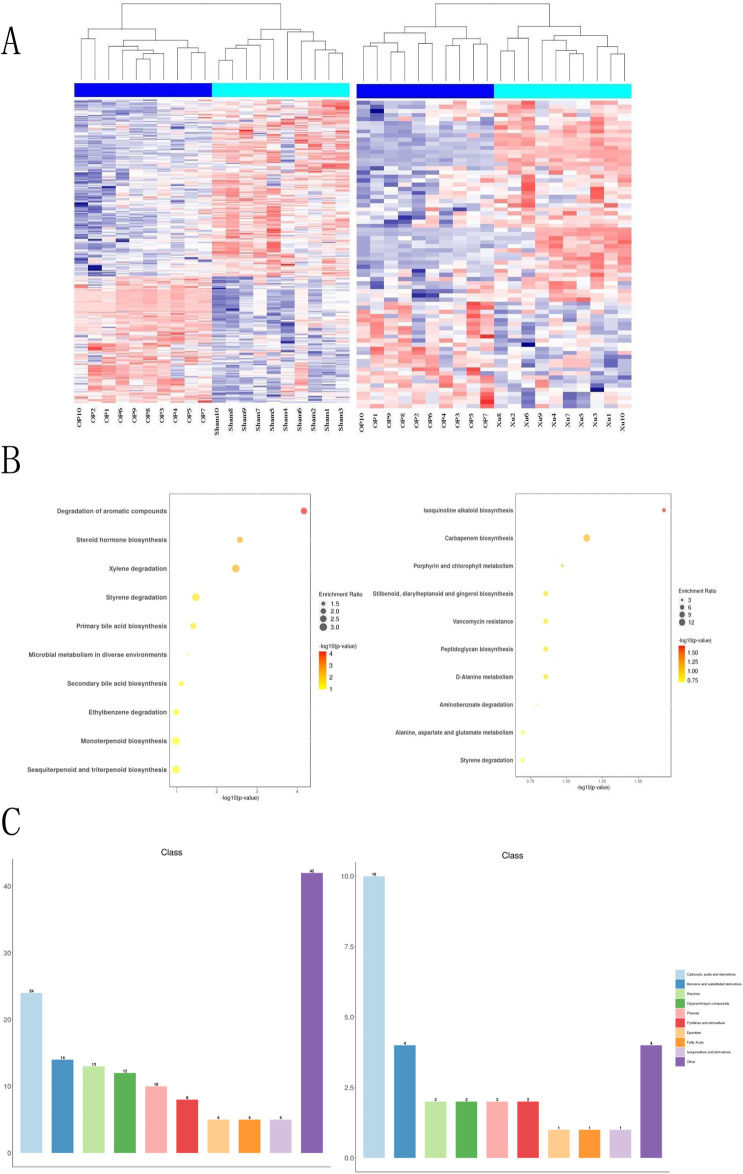
Effect of XJLGR on endogenous metabolites in plasma of OVX rats. **(A)** Hierarchical clustering heat map between groups. **(B)** Annotation by KEGG pathway enrichment top 10 between groups. **(C)** Annotation by HMDB class level histogram between groups.

This study analyzed the effects of XLJGR on endogenous plasma metabolites in OVX rats using the HPLC-MS/MS platform. A hierarchical clustering heatmap illustrated the changes in serum metabolite levels identified through positive and negative ion modes across different groups (Figure 3). The horizontal and vertical axes represent samples and differential metabolite information, respectively. Significant differences were observed between the OP and Sham groups, indicating metabolic changes in OVX rats; the Xu group also showed notable differences from the OP group, suggesting the regulatory effects of traditional Chinese medicine on OVX rat metabolism. Metabolic reactions in organisms often involve complex pathways and networks formed by various genes and proteins, leading to systematic changes in the metabolome. Therefore, we annotated and analyzed the data using KEGG and HMDB databases. KEGG enrichment analysis revealed that the most significantly altered metabolic pathway between the OP and Sham groups was the degradation of aromatic compounds, as well as differences related to steroid hormone biosynthesis and microbial metabolism in diverse environments. The pathway showing the greatest difference between the OP and Xu groups was isoquinoline alkaloid biosynthesis, with additional links to carbapenem biosynthesis and vancomycin resistance. Furthermore, the use of XLJGR led to changes in amino acid metabolism pathways, including D-alanine metabolism and alanine, aspartate, and glutamate metabolism (Figure 3B). HMDB database annotations indicated associations with steroids, carboxylic acids, fatty acyls, and prenol lipids (Figure 3C).

### 3.6 Effects of differential metabolites on MC3T3-E1 cells

**FIGURE 4:**
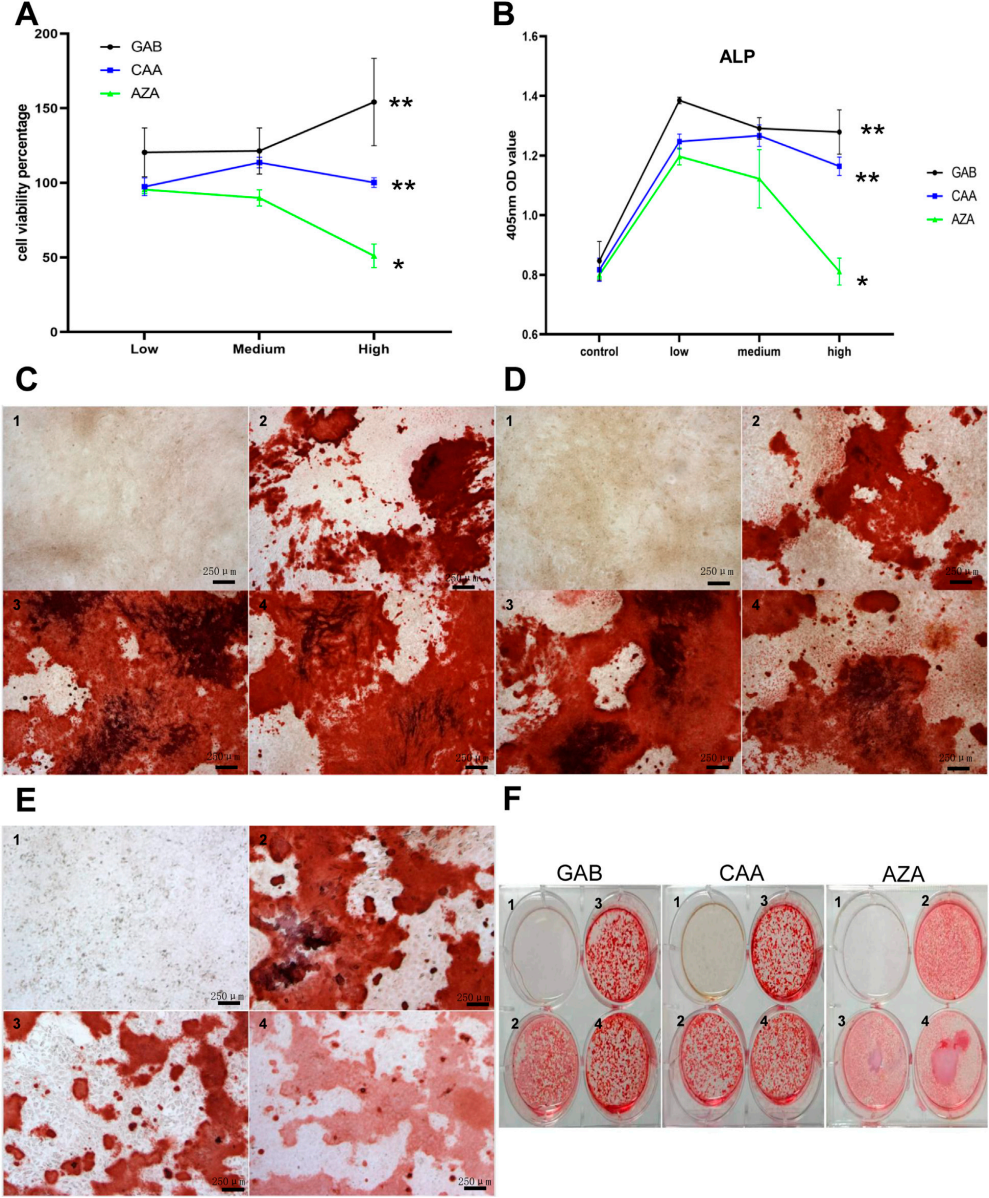
Effects of differential metabolites on MC3T3-E1 cells. **(A)** The effect of differential metabolites on the viability percentage of M3T3-E1 cells (72 h) by CCK-8 Kit. **(B)** MC3T3-E1 cells at 450 nm OD after differential metabolites intervention and ALP staining. C to F show the results of Alizarin Red S staining after intervention with different differential metabolites. **(C)** GAB. **(D)** CAA. **(E)** AZA. **(F)** Overall. (1–4 in pictures mean:control,low,medium, high group, three biological replicates on each genotype, Scale bar = 250 um).

After conducting a thorough analysis of hundreds of detected differential metabolites, we identified 36 metabolites that showed significant changes among the three groups under positive ion mode and 38 under negative ion mode. The detailed results are shown in Supplementary Table S7. According to the earlier detection of bone metabolism indicators, it is suggested that the serum level of P1NP is elevated in Xu group. After filtering through p-value and VIP value, GAB, CAA and AZA were used for the intervention on MC3T3-E1 cells. The optimal concentration of intervention was determined using the CCK-8 method. Figure 4A shows the cell survival rates for various concentrations of different metabolites after 72 h of intervention. The results indicated that different concentrations of GAB and CAA could promote the proliferation of MC3T3-E1 cells, while a higher concentration of AZA had the opposite effect. Figure 4B depicts the OD value at 405 nm under ALP staining in each group after 7 days of intervention. Except for the high concentration of AZA, all different metabolites could increase the levels of ALP in MC3T3-E1 cells. Results shown in Figures 4C–F indicate that the three interventions can enhance the mineralization ability of MC3T3-E1 cells.

## 4 Discussion

XLJGR is an empirical traditional Chinese medicine formula developed by our research team for the treatment of osteoporosis.The preliminary findings of this study provide new insights into the effects of XLJGR on intestinal microflora and endogenous metabolites in OVX rats. The gastrointestinal tract of organisms is colonized by numerous diverse microbial communities, which are considered to be the key factors for metabolic disorders (Parks et al., 2014; Li et al., 2016). In addition to the examination of the intestinal microbial composition and diversity, our study also analyzed the changes in endogenous metabolites, which further support the effects of XLJGR on the intestinal microflora of OVX rats. The enrichment results of KEGG metabolic pathway showed that “microbial metabolism in diverse environments” was significantly different between the OP and Sham groups. After treatment with XLJGR, “peptidoglycan biosynthesis”, “carbapenem biosynthesis”, “vancomycin resistance” and other pathways were altered significantly. Peptidoglycan is a primary constituent of bacterial cell membranes, and modifications in its related pathways are likely to affect the abundance of intestinal microflora. The alteration of the carbapenem and vancomycin resistance pathways may also lead to changes in antibiotic levels, which could be a contributing factor to the changes observed in the species and abundance of intestinal microflora.

Emerging research suggests that the microbiome plays a significant role in bone loss associated with estrogen deficiency (Xu et al., 2017). Other studies have demonstrated a close relationship between host immunity and bone loss in PMO, which can be influenced by the intestinal microbiome. The gut microbiota ecology is considered an important factor in energy metabolism and immune response to various diseases (Boulange et al., 2016; Niamat et al., 2024). Based on the results of metagenomic sequencing and statistical analysis, it appeared that XLJGR could alter the composition of intestinal microbiota. It was also found that XLJGR effectively alleviated PMO in rats, and its effects on the intestinal microbiota may play a role in this improvement. XLJGR significantly reduced the relative abundances of *Bacteroides* and Butyricimonas_virosa and other classified strains of intestinal bacteria. In addition, the relative abundances of Verrucomicrobium, Lachnospira, Methanobacterium, Akkermansia muciniphila, Romboutsia and other classified strains, as well as the F/B ratio, were significantly increased. Previous research has suggested that changes in the abundance of these bacterial species may be associated with osteoporosis. *Bacteroides* are more abundant in PMO and postmenopausal osteopenia groups (Lau et al., 2022). Butyricimonas_virosa is the only known strain in the Butyricimonas genus that can lead to human infection (Yuan et al., 2022). Mean F/B ratio is significantly reduced in OVX mice with osteoporosis (Sun et al., 2021). The Verrucobacteria in blank control _70 days were significantly higher than those in OVX rats_70 d (Li et al., 2019). A clinical study showed that the abundance of Spirillaceae was decreased in individuals with low BMD and was positively correlated with BMD and T scores (Liu et al., 2021). Direct supplementation of Akkermansia muciniphila is sufficient to correct the imbalance in bone metabolism induced by ovariectomy and prevent osteoporosis (Zafar and Saier, 2021). Specific bacteria, such as short-chain fatty acid (SCFA) producers (e.g., Faecalibacterium prausnitzii) and bile acid regulators (e.g., *Bacteroides* spp.), significantly influence bone metabolism. SCFAs enhance osteoblast differentiation and inhibit osteoclastogenesis, promoting bone formation. Bile acid-producing bacteria improve calcium absorption and modulate gut hormones, further supporting bone health. Dysbiosis in these microbial populations is linked to osteoporosis (Sun et al., 2021; Feng et al., 2024).

The gut microbiota-bone axis (Wang et al., 2022) refers to the complex relationship between the metabolic products of the gut microbiome and the physiological and pathological conditions of the bone. There is substantial evidence indicating that changes in the gut microbiome can affect bone strength and the mechanical properties of bone tissue (Guss et al., 2017).The results of this study found significant differences between the OP group and the Sham group, with a notable reduction in differences between the Xu group and the OP group. This indicates that the Xu Ling Jian Gu Fang has a significant regulatory effect on the plasma metabolites of PMO model rats. The mechanism is related to the regulation of the following metabolic pathways: downregulating the steroid hormone biosynthesis pathway, diversifying environmental microbial metabolism, and degrading aromatic compounds in the OP group. The XLJRG upregulated key metabolites such as GBP, CAA, and AZA in the OP group, as well as significantly upregulating metabolic pathways related to peptidoglycan biosynthesis, carbapenem biosynthesis, vancomycin resistance, and other metabolic pathways.

AZA is a small molecule that scavenges intracellular reactive oxygen species (ROS) and free radicals, demonstrating antitumor effects on various cancer cells. It has been shown to lower ROS levels and enhance the antioxidant capacity in acute myeloid leukemia cell lines and patient cells (Zhang et al., 2020). Elevated ROS levels are associated with diabetic osteoporosis (Schroder, 2015), where excessive ROS accumulation can reduce antioxidant enzyme levels and inhibit osteoblast differentiation, leading to bone loss (Tao et al., 2020). Our experiment indicates that low to moderate concentrations of AZA increase ALP levels in MC3T3-E1 cells by reducing intracellular ROS. Research (Duttenhoefer et al., 2014) showed that osteoblasts *in vitro* stained significantly with Alizarin Red by day 7; calcification induced by novel derivatives only occurred at nanomolar concentrations (Lee et al., 2010). found that on day 15 of differentiation, continuous treatment with camphoric acid (up to 5 μM) accelerated calcium formation, while day 18 staining revealed mineralization induced by low camphoric acid concentrations. Since CAA showed a stimulatory peak between 2.5 and 5 μM and an inhibitory trend at 10 μM, low doses may promote osteoblast differentiation by inducing the expression of glutamate signaling molecules (NMDAR1, GluR3, GluR4, and mGluR8) in mouse osteoblasts. GABA and its metabolites influence peptidoglycan biosynthesis by regulating gut microbiota and cellular signaling pathways, promoting cell proliferation and repair. Additionally, GABA’s stimulatory effect on osteoblasts and inhibitory effect on osteoclasts help maintain bone density. Its anti-inflammatory properties also contribute to reducing the risk of osteoporosis (Arachchilage et al., 2023).

Based on previous studies and multi-omics analysis, this study explored the gut microbiota-bone axis mechanism of XLJRG. However, it should be noted that this study had some limitations. Due to cost considerations, a positive control group was not included, and the sample size was small. As a result, the conclusions drawn from this study were mostly descriptive and speculative, and should be interpreted with caution. Further research with larger sample sizes and appropriate control groups may be necessary to confirm the findings of this study and to better understand the bone immune mechanism of XLJRG. Despite these limitations, this study provides novel insights into the potential mechanisms underlying the therapeutic effects of XLJRG in osteoporosis, and could inform future research in this area.

## 5 Conclusion

In summary, XLJGR can improve BMD in OVX rats, and its gut microbiota-bone axis mechanism may be related to changes in the abundance of intestinal microflora and the composition of endogenous metabolites. These findings suggest that XLJGR may modulate the gut microbiota and affect the metabolism of endogenous compounds, leading to improvements in bone health. However, further research is needed to fully understand the mechanisms involved and to confirm the therapeutic potential of XLJGR in the treatment of osteoporosis.

## Data Availability

The original contributions presented in the study are included in the article/Supplementary Material, further inquiries can be directed to the corresponding author.

## Abbreviations

GM, gut microbiota; LEfSe, linear discriminant analysis combined with effect-size measurements; OP, osteoporosis; XLJGR, xuling jiangu recipe.
