# Supplementary material for: Herbal formula xuling-jiangu improves bone metabolic balance in rats with ovariectomy-induced osteoporosis via the gut-bone axis
Source: Front Pharmacol. 2024 Nov 13;15:1505231. doi: 10.3389/fphar.2024.1505231 (PMC11598424; doi:10.3389/fphar.2024.1505231)
Supplement: Supplementary file 1 [file Table1.pdf]

Supplementary Data

Table S1. The composition of Xu-Ling-Jian-Gu Recipe

Chinese name	Scientific name	Weight
Chuan Xu Duan	*Dipsacus asperoides C. Y. Cheng et T. M. Ai*	15g
Gu Sui Bu	*Davallia mariesii Moore ex Bak.*	15g
Nv Zhen Zi	*Fructus Ligustri Lucidi*	15g
Fu Ling	*Poria cocos(Schw.)Wolf*	12g
Chi Shao	*Paeonia lactiflora Pall.*	10g
Yan Hu Suo	*Corydalis yanhusuo W. T. Wang*	10g
Mu Dan Pi	*Paeonia × suffruticosa Andr.*	10g
Chuan Xiong	*Ligusticum chuanxiong hort*	10g
Hong Hua	*Carthamus tinctorius L.*	9g
Bai Zhu	*Atractylodes macrocephala Koidz.*	8g
Chen Pi	*Citrus reticulata Blanco*	5g
Gan Cao	*Glycyrrhiza uralensis Fisch.*	3g

Table S2. The calibration curves, correlation coefficients,retention time(RT) of XLJGR effective components.

Effective components	Calibration curves	R^2	RT(min)
Gallic acid	y = 12.5246 x - 0.5632	0.9998	6.247
Hydroxy safflower yellow pigment A	y = 12.4075 x + 1.0744	0.9997	18.193
Paeoniflorin	y = 12.9552 x - 2.1195	0.9998	29.307
Ferulic acid	y = 45.5390 x - 2.0578	0.9999	36.483
Naringin	y = 39.8583 x + 0.6437	0.9996	46.743
Tetrahydropalmatine	y = 67.9694 x - 0.6477	0.9994	48.037
Dehydrogenated pachymic acid	y = 1.9528 x - 1.3990	0.9998	56.477
Atractylone	y = 0.5025 x - 0.0398	0.9997	57.240
Dipsacoside VI	y = 1.9913 x + 0.9766	0.9994	59.957
Paeonol	y = 38.7890 x - 2.5849	0.9998	62.197

Table S3. Comparison of bone mineral density of tibia among groups (x ±SD, g/cm^2)

Group	N	BMD
OP	10	0.259±0.010
Sham	10	0.271±0.011*
Xu	10	0.267±0.009*

Notes: * Compare with OP group,$P < 0.05$

Table S4. Comparison of bone metabolism related indexes of rats in each group(x ±SD, pg/ml)

Group	CTX-1	P1NP
OP	29.78±2.11[#]	1129.75±157.76
Sham	37.21±4.95	1602.06±353.09[*]
Xu	29.14±0.87[#]	1699.88±559.17

Notes: [*] Compare with OP group, $P < 0.05$; [#] Compare with Sham group, $P < 0.05$

Table S5. Top 10 differential expression genes between OP vs. Sham and Xu vs. OP groups by GO analysis

Differentially expressed genes	logFC	adj.P.Val	up/down	group
cellular alkane metabolic process	2.218	0.000011	up	1
methane metabolic process	2.340	0.000012	up	1
presynaptic membrane	4.019	0.000012	up	1
sodium channel activity	4.013	0.000012	up	1
neuron projection cytoplasm	4.013	0.000012	up	1
exocytic vesicle membrane	4.013	0.000012	up	1
kainate selective glutamate receptor activity	4.013	0.000012	up	1
synaptic vesicle membrane	4.013	0.000012	up	1
terminal bouton	4.013	0.000012	up	1
regulation of synaptic transmission, glutamatergic	4.013	0.000012	up	1
glycerol-3-phosphate dehydrogenase complex	-6.703	0.000009	down	1
sn-glycerol-3-phosphate:ubiquinone oxidoreductase activity	-6.703	0.000009	down	1
histidine transport	-6.675	0.000009	down	1
histidinol-phosphate transaminase activity	-6.262	0.000009	down	1
2-dehydro-3-deoxy-6-phosphogalactonate aldolase activity	-7.009	0.000009	down	1
regulation of FtsZ-dependent cytokinesis	-6.123	0.000009	down	1
negative regulation of FtsZ-dependent cytokinesis	-6.123	0.000009	down	1
regulation of asexual reproduction	-6.123	0.000009	down	1
negative regulation of asexual reproduction	-6.123	0.000009	down	1
23S rRNA (adenine(1618)-N(6))-methyltransferase activity	-6.273	0.000009	down	1
5,6,7,8-tetrahydromethanopterin metabolic process	4.049	0.004049	up	2
methanopterin-containing compound metabolic process	4.049	0.004049	up	2
methanopterin-containing compound biosynthetic process	4.049	0.004049	up	2
tetrahydromethanopterin biosynthetic process	4.049	0.004049	up	2
5,6,7,8-tetrahydromethanopterin biosynthetic process	4.049	0.004049	up	2
tetrahydromethanopterin metabolic process	4.049	0.004049	up	2
ribonuclease MRP complex	3.532	0.008953	up	2
steroid biosynthetic process	3.774	0.008953	up	2
LPPG:FO 2-phospho-L-lactate transferase activity	3.724	0.010769	up	2
GMP binding	3.707	0.012987	up	2
glucosamine-6-phosphate deaminase activity	-2.313	0.008953	down	2
dihydrofolate metabolic process	-1.252	0.012118	down	2
dihydrofolate synthase activity	-1.259	0.012118	down	2
tetrahydrofolylpolyglutamate biosynthetic process	-1.259	0.012118	down	2
tetrahydrofolylpolyglutamate synthase activity	-1.259	0.012118	down	2
dihydrofolate biosynthetic process	-1.259	0.012118	down	2
tetrahydrofolylpolyglutamate metabolic process	-1.259	0.012118	down	2
signal recognition particle	-1.058	0.012987	down	2
pyrimidine-containing compound transmembrane transport	-1.091	0.016084	down	2
DNA topoisomerase type I activity	-1.876	0.017612	down	2

Notes:group 1: OP vs. Sham; group 2: Xu vs. OP.

TableS 6. Top 5 differential expression genes between OP vs. Sham and Xu vs. OP groups by KEGG analysis

Differentially expressed genes	logFC	adj.P.Val	up/down	group
Endocrine resistance	2.730	0.016743	up	1
Acute myeloid leukemia	2.722	0.016743	up	1
Fc gamma R-mediated phagocytosis	2.722	0.016743	up	1
TGF-beta signaling pathway	2.722	0.016743	up	1
ErbB signaling pathway	2.722	0.016743	up	1
Betalain biosynthesis	-5.495	0.000331	down	1
Geraniol degradation	-3.456	0.000365	down	1
Fluorobenzoate degradation	-3.508	0.000993	down	1
Chemical carcinogenesis	-4.199	0.000993	down	1
Ethylbenzene degradation	-5.970	0.001339	down	1
Carotenoid biosynthesis	1.382	0.011884	up	2
Cell cycle	3.031	0.019682	up	2
Flavone and flavonol biosynthesis	1.099	0.022657	up	2
Fanconi anemia pathway	3.178	0.026022	up	2
Non-homologous end-joining	1.145	0.026022	up	2
Glycosphingolipid biosynthesis - ganglio series	-1.242	0.011884	down	2
MAPK signaling pathway - fly	-1.084	0.011884	down	2
Acute myeloid leukemia	-3.450	0.011884	down	2
Fc gamma R-mediated phagocytosis	-3.450	0.011884	down	2
TGF-beta signaling pathway	-3.450	0.011884	down	2

Notes::group 1: OP vs. Sham; group 2: Xu vs. OP.

Table S7. Identification results of differential metabolites among groups

ion mode	Name	Formula	Molecular Weight	Retention time(min)	OP vs. Sham			Xu vs. OP		
					VIP	FC	P	VIP	FC	P
+	I-Urobilinogen	$C_{33}H_{44}N_4O_6$	592.3260	6.181	1.422348	6.200781	0.000775	1.903152	0.276065	0.014433
+	Phenacetin	$C_{10}H_{13}NO_2$	179.0946	8.109	1.410666	2.249923	0.002594	1.299885	1.303285	0.046543
+	N-Ethylpentylone	$C_{14}H_{19}NO_3$	249.1366	2.017	1.087327	0.635316	0.016151	2.805082	109.891535	0.000000
+	tilisolol	$C_{17}H_{24}N_2O_3$	304.1788	8.090	1.223913	1.935874	0.015533	1.491340	1.449577	0.015805
+	N-Acetyl-L-citrulline	$C_8H_{15}N_3O_4$	217.1064	1.275	1.023633	0.394546	0.035056	1.505140	1.974484	0.047495
+	MDMA Methylene homolog	$C_{12}H_{17}NO_2$	207.1262	3.295	1.060996	1.391722	0.021196	1.655678	1.461635	0.015554
+	[FA(16:2)]N-hexadecyl-ethanolamine	$C_{18}H_{37}NO_2$	299.2824	9.119	1.320599	2.486503	0.004742	1.629017	0.602354	0.028810
+	Gabaculine	$C_7H_9NO_2$	139.0634	0.654	1.587832	0.539054	0.000029	1.554995	1.356448	0.034696
+	4beta-methylzymosterol-4-carbaldehyde	$C_{29}H_{46}O_2$	426.3500	9.078	1.482005	0.164650	0.000453	1.528305	1.893399	0.046446
+	Dinaciclib	$C_{21}H_{28}N_6O_2$	396.2255	7.694	1.496723	0.031940	0.000144	1.547120	2.496889	0.028659
+	2762577	$C_{15}H_{23}N_3O_4$	309.1690	1.841	1.270636	4.616904	0.003939	1.562830	0.271807	0.011923
+	Ecgonine	$C_9H_{15}NO_3$	185.1054	0.636	1.048818	0.708133	0.046830	1.442897	1.302412	0.046381
+	Hexanoylglycine	$C_8H_{15}NO_3$	173.1054	0.663	1.530326	0.465095	0.000156	1.650218	1.462546	0.021932
+	3-Methylcrotonylglycine	$C_7H_{11}NO_3$	157.0740	0.635	1.374064	0.524318	0.002760	1.769427	1.748784	0.012902
+	Isoxaben	$C_{18}H_{24}N_2O_4$	332.1736	5.585	1.326961	4.423674	0.002220	1.804920	0.343120	0.016630
+	N5-Ethyl-L-glutamine	$C_7H_{14}N_2O_3$	174.1006	0.630	1.073772	0.539358	0.046812	1.432976	1.865318	0.044629
+	?-(Methylenecyclopropyl)glycine	$C_6H_9NO_2$	127.0636	4.517	1.104694	0.626361	0.039100	1.550222	2.352641	0.032147
+	N-Methylphenylethanolamine	$C_9H_{13}NO$	151.0998	1.553	1.109067	0.674077	0.023621	2.086256	8.568021	0.000626
+	Methohexital	$C_{14}H_{18}N_2O_3$	262.1320	4.201	1.156466	2.179677	0.005662	1.408777	0.586058	0.048962
+	Spiroxamine	$C_{18}H_{35}NO_2$	297.2669	8.702	1.112779	1.687052	0.025045	1.516448	0.519064	0.036569
+	1_6_6-Trimethyl-2_7-dioxabicyclo[3.2.2]nonan-3-one	$C_{10}H_{16}O_3$	184.1101	3.325	1.230659	0.605079	0.003997	2.126176	6.155013	0.000364
+	2-Hydroxy-N-(2-hydroxyethyl)propionamide	$C_5H_{11}NO_3$	133.0742	0.639	1.229607	0.529159	0.004715	1.789716	1.892450	0.002768
+	Cerulenin	$C_{12}H_{17}NO_3$	223.1209	1.505	1.167747	0.314942	0.027021	2.274487	3.263316	0.000049
+	Benzaldehyde	C_7H_6O	106.0423	4.153	1.469947	4.612683	0.000403	1.633851	0.427066	0.021792
+	N-({(2R,4S,5R)-5-[6-(2-Furyl)-2-methyl-4-pyrimidinyl]-1-azabicyclo[2.2.2]oct-2-yl}methyl)-2-(2-thienyl)acetamide	$C_{23}H_{26}N_4O_2S$	422.1779	1.455	1.074306	0.085214	0.027749	1.853751	26.100562	0.006448
+	Cede+fingol	$C_{20}H_{41}NO_3$	343.3088	9.252	1.248765	2.155666	0.006956	1.996650	0.655977	0.002864
+	Gabapentin	$C_9H_{17}NO_2$	171.1261	1.836	1.025542	0.663041	0.037415	2.558502	7.490131	0.000003
+	Dibutylone	$C_{13}H_{17}NO_3$	235.1210	1.400	1.354290	0.475253	0.003425	2.230157	17.359794	0.000188
+	Phenylacetaldehyde	C_8H_8O	120.0578	4.175	1.465034	4.356468	0.000434	1.710217	0.408403	0.016866

+	Gly-Phe	$C_{11}H_{14}N_2O_3$	222.1006	4.177	1.182637	0.209964	0.014306	1.996372	3.041114	0.001978
+	N-[(S)-(+)-1-Ethoxycarbonyl-3-phenylpropyl]-L-alanine	$C_{15}H_{21}NO_4$	279.1471	1.524	1.415504	0.468147	0.001519	2.154929	16.269031	0.000401
+	Coniferylalcohol	$C_{10}H_{12}O_3$	180.0788	3.519	1.501592	0.305437	0.000387	2.710574	10.875159	0.000000
+	16-feruloyloxypalmitic acid	$C_{26}H_{40}O_6$	448.2831	6.765	1.560606	0.130493	0.000227	1.555365	2.313112	0.013320
+	(S)-N-Methylcanadine	$C_{21}H_{23}NO_4$	353.1628	6.067	1.082165	6.293055	0.026164	1.362947	1.593578	0.042150
+	Minoxidil	$C_9H_{15}N_5O$	209.1278	1.830	1.079657	0.340080	0.045141	1.477325	1.921833	0.031666
-	18-acetoxy-1alpha-hydroxyvitamin D3	$C_{29}H_{46}O_4$	458.3404	8.930	1.084781	0.364622	0.036547	1.469025	1.613855	0.025207
-	[ST(3:0)]5beta-Cholestane-3alpha_7alpha_12alpha-triol	$C_{27}H_{48}O_3$	420.3611	13.451	1.190993	0.707933	0.014446	1.622074	1.415334	0.009500
-	7-Sulfocholic acid	$C_{24}H_{40}O_8S$	488.2450	7.135	1.248447	1.283894	0.005491	1.581472	0.283885	0.006889
-	7-O-Methylluteone	$C_{21}H_{20}O_6$	368.1268	9.956	1.429507	0.104792	0.000656	1.662434	2.810854	0.003711
-	I-Urobilinogen	$C_{33}H_{44}N_4O_6$	592.3272	6.219	1.495721	6.244862	0.000289	1.366174	0.356709	0.026158
-	2-Oxooctadecanoicacid	$C_{18}H_{34}O_3$	298.2512	11.835	1.298427	1.909308	0.007267	1.356689	0.571332	0.029563
-	LicoisoflavoneA	$C_{20}H_{18}O_6$	354.1112	8.311	1.164713	0.095452	0.009873	1.422893	4.549465	0.012005
-	Cortolone	$C_{21}H_{34}O_5$	366.2414	5.649	1.465943	0.022380	0.000317	1.504674	2.245211	0.040783
-	Azelaic acid	$C_9H_{16}O_4$	188.1044	2.956	1.372485	0.618050	0.001731	2.464675	20.889838	0.000000
-	[6]-Gingerol	$C_{17}H_{26}O_4$	294.1835	13.081	1.424070	0.341131	0.000629	1.319277	1.571052	0.026635
-	3-Hydroxyaminophenol	$C_6H_7NO_2$	125.0472	3.241	1.495061	0.503856	0.000357	1.441839	1.335456	0.047183
-	butralin	$C_{14}H_{21}N_3O_4$	295.1538	5.812	1.556494	26.430504	0.000085	1.912891	0.065430	0.000310
-	Eglumetad	$C_8H_{11}NO_4$	185.0684	3.256	1.492931	0.507288	0.000377	1.446684	1.333430	0.046992
-	2,3,14,20-Tetrahydroxy-22,23-epoxyergost-7-en-6-one	$C_{28}H_{44}O_6$	476.3146	7.402	1.446894	2.207311	0.001181	1.693305	0.588728	0.008410
-	Pyridoxine	$C_8H_{11}NO_3$	169.0733	1.236	1.798380	0.090219	0.000000	1.727211	1.943692	0.004265
-	(+/-)-Camphoric acid	$C_{10}H_{16}O_4$	200.1050	4.651	1.281265	0.575334	0.005353	2.117066	19.340144	0.000125
-	Azelaic acid	$C_9H_{16}O_4$	188.1046	2.309	1.333669	0.614904	0.003668	2.073999	4.408292	0.000372
-	3-(Sulfooxy)benzenepropanoic acid	$C_9H_{10}O_6S$	246.0200	3.191	1.276826	0.020963	0.008663	1.732991	1.792562	0.004791
-	Meticrane	$C_{10}H_{13}NO_4S_2$	275.0292	3.314	1.044978	2.967427	0.037427	1.651569	2.489154	0.010378
-	1-(2-Furylmethyl)-5-oxopyrrolidine-3-carboxylic acid	$C_{10}H_{11}NO_4$	209.0686	1.656	1.591171	0.271546	0.000023	1.676219	1.861685	0.004441
-	Gabaculine	$C_7H_9NO_2$	139.0628	0.728	1.272978	0.536220	0.017693	1.438819	1.332722	0.046456
-	Azelaicacid	$C_9H_{16}O_4$	188.1047	3.489	1.390908	0.330131	0.002455	1.632016	1.951664	0.005176
-	N5-(L-1-Carboxyethyl)-L-ornithine	$C_8H_{16}N_2O_4$	204.1107	1.065	1.026963	1.879360	0.018543	1.333209	0.580509	0.046601
-	3-Hydroxy-4-methylanthranilate	$C_8H_9NO_3$	167.0577	1.234	1.515413	0.527650	0.000268	1.388552	1.342311	0.044026
-	[FAhydroxy(4:0)]N-(3S-hydroxy-	$C_8H_{13}NO_4$	187.0840	1.119	1.338792	0.446145	0.002508	1.655347	1.578758	0.013636

	butanoyl)-homoserinelactone									
-	(1S,4aR,5S)-5-[(3E)-5-Methoxy-3-methyl-5-oxo-3-penten-1-yl]-1,4a-dimethyl-6-methylenedecahydro-1-naphthalenecarboxylic acid	$C_{21}H_{32}O_4$	348.2307	9.287	1.449306	2.952618	0.000846	1.334602	1.324077	0.046108
-	3-Hydroxysebacic acid	$C_{10}H_{18}O_5$	218.1153	2.156	1.151200	0.584057	0.049836	2.367858	10.218843	0.000001
-	MFCD00270409	$C_{21}H_{38}O_4$	354.2778	9.983	1.102164	2.869451	0.027234	1.498750	2.389664	0.011223
-	5-Nitroindoline	$C_8H_8N_2O_2$	164.0579	1.297	1.274365	0.631229	0.009304	1.566990	1.429957	0.021638
-	2-(5'-methylthio)pentylmalate	$C^{10}H^{18}O^5S$	250.0878	3.315	1.297011	0.452852	0.008153	1.957998	9.620144	0.000123
-	15(S)-HpEDE	$C^{20}H^{36}O^4$	340.2616	8.650	1.368431	2.310178	0.001759	1.452697	1.761029	0.017502
-	gamma-Glutamyl-gamma-aminobutyraldehyde	$C^9H^{16}N^2O^4$	216.1108	1.138	1.115886	1.620682	0.013781	1.370693	0.584981	0.026316
-	Norepinephrine(Noradrenaline)	$C^8H^{11}NO^3$	169.0732	0.744	1.648170	0.289658	0.000008	1.674479	1.352369	0.022218
-	NJ5000000	$C^8H^{15}N^3O^2$	185.1160	1.354	1.424210	0.498008	0.002067	2.022085	3.682720	0.000177
-	2-O-ETHYL ASCORBIC ACID	$C^8H^{12}O^6$	204.0632	0.718	1.177794	0.592361	0.017642	1.583035	1.368829	0.029346
-	3-Hydroxy-4-methylanthranilate	$C_8H_9NO_3$	167.0579	0.683	1.244649	0.612369	0.005898	1.537698	1.391834	0.020581
-	Ptaquiloside	$C_{20}H_{30}O_8$	398.1948	5.128	1.268342	0.525511	0.005872	2.269636	9.774153	0.000006
